# Insufficient documentation for clinical efficacy of selenium supplementation in chronic autoimmune thyroiditis, based on a systematic review and meta-analysis

**DOI:** 10.1007/s12020-016-1098-z

**Published:** 2016-09-28

**Authors:** Kristian Hillert Winther, Johanna Eva Märta Wichman, Steen Joop Bonnema, Laszlo Hegedüs

**Affiliations:** 1 Department of Endocrinology and Metabolism, Odense University Hospital, Kløvervænget 10, 6th floor, Odense C, 5000 Denmark; 2 Department of Clinical Research, Faculty of Health Sciences, University of Southern Denmark, Winsløwparken 19, 3. Sal, Odense C, 5000 Denmark

**Keywords:** Chronic autoimmune thyroiditis, Hashimoto’s thyroiditis, Selenium supplementation, Thyroid hormones, Systematic review, Meta-analysis, Quality of life, Thyroid ultrasound

## Abstract

By a systematic review and meta-analysis to investigate clinically relevant effects of selenium supplementation in patients with chronic autoimmune thyroiditis. Controlled trials in adults (≥18 years) with autoimmune thyroiditis, comparing selenium with or without levothyroxine substitution, versus placebo and/or levothyroxine substitution, were eligible for inclusion. Identified outcomes were serum thyrotropin (thyroid stimulating hormone) levels in LT4-untreated patients, thyroid ultrasound and health-related quality of life. Eleven publications, covering nine controlled trials, were included in the systematic review. Random effects model meta-analyses were performed in weighted mean difference for thyroid stimulating hormone, ultrasound and health-related quality of life. Quality of evidence was assessed per outcome, using GRADE. Meta-analyses showed no change in thyroid stimulating hormone, or improvements in health-related quality of life or thyroid echogenicity (ultrasound), between levothyroxine substitution-untreated patients assigned to selenium supplementation or placebo. Three trials found some improvement in wellbeing in patients receiving levothyroxine substitution, but could not be synthesized in a meta-analysis. The quality of evidence ranged from very low to low for thyroid stimulating hormone as well as ultrasound outcomes, and low to moderate for health-related quality of life, and was generally downgraded due to small sample sizes. We found no effect of selenium supplementation on thyroid stimulating hormone, health-related quality of life or thyroid ultrasound, in levothyroxine substitution-untreated individuals, and sporadic evaluation of clinically relevant outcomes in levothyroxine substitution-treated patients. Future well-powered RCTs, evaluating e.g. disease progression or health-related quality of life, are warranted before determining the relevance of selenium supplementation in autoimmune thyroiditis.

## Introduction

Chronic autoimmune (AIT) or Hashimoto’s thyroiditis affects 1–2 % of the population with increasing prevalence with age and a female preponderance. In communities replete in iodine intake, it is the predominant cause of hypothyroidism [1, 2]. The etiology is multifactorial and based on genetic susceptibility in a complex interaction with numerous environmental triggers [3, 4], possibly including selenium deficiency [5]. No cure exists, and the standard treatment is life-long levothyroxine substitution (LT4) to normalize circulating thyrotropin [thyroid stimulating hormone (TSH)] levels. Recent insights suggest that LT4 cannot ensure a euthyroid state in all tissues simultaneously [6], and a place for selenium supplementation in the treatment of AIT has been much debated [7]. Selenium is an essential micronutrient with a wide range of effects in, e.g. redox homeostasis, immunity, and thyroid hormone metabolism [8]. Since 2002, a number of trials have investigated the effects of selenium supplementation in AIT. In a meta-analysis from 2010, based on four trials, [9], the authors reported decrease in thyroid peroxidase autoantibody (TPO-Ab) levels and improvement in well-being and/or mood, after 3 months of selenium supplementation, as compared to placebo. Similar conclusions were reached in another meta-analysis from 2014, including nine trials [10]. Finally, a Cochrane Collaboration systematic review from 2013, also including four studies, [11] reached no conclusion regarding effects on HRQL, and did not perform a meta-analysis of change in TPO-Ab because of considerable heterogeneity among the included studies. While TPO-Ab levels are central to the diagnosis of AIT [12], their clinical importance is less clear once treatment is initiated. The GRADE guidelines provide a framework for determining outcomes of interest [13], and rate morbidity or disease remission as crucial when evaluating treatment effects [13]. However, none of these outcomes were reported in the previous systematic reviews [9–11]. Further, the safety of upper tolerable selenium intake limits has been questioned [14], underlining that supplementation should only be administered to correct deficits or on solid clinical indication.

Due to insufficient trial evaluation of clinically relevant outcomes, and in view of recent safety concerns, we hypothesized that selenium supplementation does not currently have a place in the treatment of AIT, and tested this hypothesis in a systematic review and meta-analysis.

## Materials and methods

### Criteria for considering studies for this review

- *Type of participants* Adults (≥18 years) with AIT
- *Types of intervention* Any dose of selenium supplementation, alone or combined with LT4.
- *Types of controls* Placebo alone, placebo combined with LT4, or no treatment.
- *Types of outcomes* Mortality, morbidity, disease progression and/or remission, LT4 dose, HRQL, thyroid function, thyroid ultrasound (US), adverse effects.
- *Types of studies* Controlled trials.

### Focused review question

Which effect does selenium supplementation have on the clinical course of AIT.

### Literature search

The following databases were searched the 23rd of September 2015: Medline (from inception until 23rd of September 2015), EMBASE (from 1974 until 22nd of September 2015) and Cochrane Central Register of Controlled Trials (CENTRAL) (from inception until 23rd of September 2015).

### Selection of studies

records were imported from PubMed, EMBASE and CENTRAL into Covidence (Covidence systematic review software, Veritas Health Innovation, Melbourne, Australia, available at www.covidence.org) where duplicates were removed. Two reviewers (KHW and JW) independently screened the remaining records, first by title and then by abstracts, using the Covidence software platform. Differences of opinion were resolved by discussion and consensus. The screening process was documented in a preferred reporting items for systematic reviews and meta-analyses (PRISMA) () flow chart of study selection.

### Data extraction

For studies that fulfilled the inclusion criteria, two review authors (JW, KW) independently extracted relevant population and intervention characteristics onto a pre-designed template. Where we may have had further questions regarding one or more of the included trials, we sent an email request to the corresponding author of the study.

### Data synthesis

Data was extracted as means with standard deviations (SD). Where data was presented as median with 95 % confidence interval (CI), an SD was calculated using the formula ((HCI-LCI)/2/TINV(0.05; *n *− 1) * sqrt (*n*)), where HCI is the highest value in the confidence interval (CI), LCI the lowest value of the CI, and *n* the sample size of the group [15]. Where data was presented as median with interquartile range (IQR), the median was used as a mean and a SD was calculated by the formula IQR/1.35 [15]. Where data was presented as median with range, SD was estimated to be one quarter of the range [15]. One trial, reported in two publications [16, 17], had four arms [selenium vs. LT4 vs. selenium + LT4 vs. placebo]. We included the selenium vs. placebo arms in the meta-analyses for effects on thyroid function in an LT4-untreated population. Meta-analyses in weighted mean difference (WMD) were performed for TSH using the follow-up scores at 3, 6 and 12 months, improvement in thyroid echogencity at 3 and 6 months, and for the generic HRQL instrument SF-36, using follow-up scores at 6 months. The SF-36 is a general health questionnaire divided into eight domains, covering different aspects of physical and mental well-being [18]. The heterogeneity of the included studies was evaluated by the *I* ^2^ statistics: 0–40 % might not be important; 30–60 % may represent moderate heterogeneity; 50–90 % may represent substantial heterogeneity; 75–100 % considerable heterogeneity. The outcomes ‘reduction in thyroid volume’ and ‘improvement in HRQL using other instruments than SF-36’, could not be assessed in meta-analyses due to insufficient data, and were assessed qualitatively.

### Quality of evidence

Quality of evidence for each outcome and risk of bias for each study was assessed independently by KHW, JW and a local consultant, using the GRADE guidelines [19].

The meta-analyses were performed in a random effects model using STATA version 13.1 (2013; Stata Corporation, College Station, TX, USA).

The systematic review was registered at PROSPERO on the 24th of August 2015. Registration number: CRD42015025247.

## Results

### Search results

**Fig. 1:**
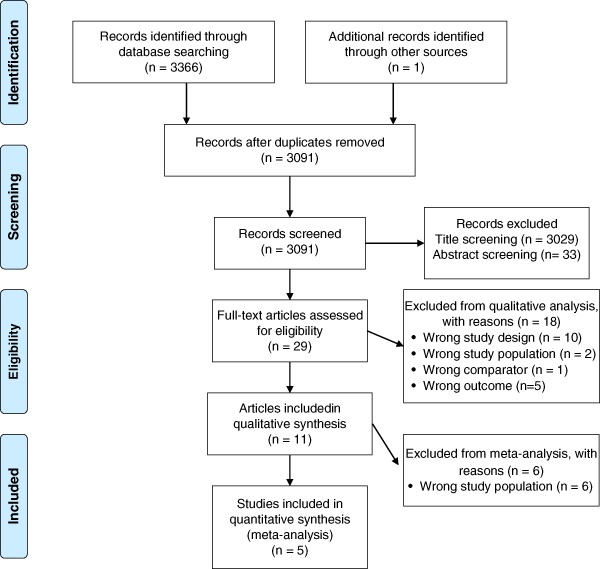
Preferred reporting items for systematic reviews and meta-analyses (PRISMA) flow chart. Flow chart of study selection process, with number of studies excluded at each step in the systematic review and meta-analysis. PRISMA flow diagram

3366 records were identified in the Medline, EMBASE and CENTRAL databases. The screening process is presented in Fig. 1. One article was identified by one of the authors from another source [20]. All records were imported into the Covidence software platform and 276 duplicates were removed. Two reviewers (JW and KW) independently screened the remaining 3091 records. 3029 records were excluded following title screening, leaving 62 records to be screened by abstract. 33 records were excluded after abstract screening, leaving 29 articles to be assessed in full-text. Full-text evaluation was carried out independently by two reviewers (KW, JW) except for one record, written in Hungarian [21], which was translated by a colleague. We excluded conference abstracts [22–26], records published in Chinese [27], and studies among pregnant women [28, 29]. Furthermore, one study was excluded due to a wrong comparator, because both intervention- and control groups received selenium supplementation [30]. Four records were excluded on the basis of wrong study design, being reviews [9–11] or a case report [31]. Finally, five trials were excluded because they only investigated effects on thyroid autoantibody levels [21, 32–35], which we disregarded in this context. Throughout the screening process, differences of opinion were resolved by discussion and consensus. In total, eleven publications reporting data from nine trials were included in the systematic review [16, 17, 20, 36–43]. In the meta-analysis on TSH, five publications were excluded because the trial populations consisted of AIT patients receiving LT4 therapy [16, 17, 36, 37, 43], and one publication was excluded [16] because data from the same trial population was reported in reference [40].

### Systematic review

The eleven publications included from the search were published between 2002 and 2015, and reported data from nine trials in 679 individuals. Identified outcomes of importance for patients and clinicians were TSH levels, HRQL and thyroid volume and echogenicity, assessed by US. Data on mortality, morbidity or disease remission were not identified. The main data are summarized in Table 1. All trials, with the exception of one from Brazil [43], were conducted in Europe. All trials used placebo as a control, except one [38] that used no treatment. Four trials, reported in five publications, were double-blinded [20, 39, 40, 42, 43]. One was single-blinded (US investigators) [38]. Two trials were described as blinded without specification [17, 36], while the follow-up study by Gartner et al. [37] was open-label, along with another trial [41]. In one trial, the authors did not account for blinding [16].

**Table 1:** Characteristics of included studies

Study	Country	Sample size (int/con)	Intervention groups	LT4 baseline	Age in years	% Female	Evaluated outcomes	Months of intervention
Pilli [20]	Italy	*N* = 60 (20/20/20)	80 μg Seme vs. 160 μg Seme vs. placebo	None	Mean: 43	100	TSH, FT3, FT4, SF-36, US: echogenicity, volume	12
De Farias [43]	Brazil	*N* = 55 (28/27)	200 μg Seme vs. placebo	Some	Median 46	91	US: echogenicity, volume	3
Eskes [42]	The Netherlands	*N* = 61 (30/31)	200 μg Na_2_SeO_3_ vs. placebo	None	Median 44.3	90	TSH, FT4, SF-36	6
Anistasilakis [41]	Greece	*N* = 86 (61/25)	200 μg Seme for 3 or 6 months vs. placebo	Some	Mean 43.7	62	TSH, FT4, FT3	3 or 6
Krysiak [40]^a^	Poland	*N* = 155 (79/76)	200 μg Seme vs. 200 μg Seme + LT4 vs. LT4 or placebo	None	Mean 40.5	100	TSH, FT4, FT3	6
Krysiak [39]^a^	Poland	*N* = 170 (86/84)	200 μg Seme vs. 200 μg Seme + LT4 vs. LT4 or placebo	None	Mean 38.5	100	TSH, FT4, FT3	6
Nacamulli [38]	Italy	*N* = 76 (46/30)	80 μg Na_2_SeO_3_ vs. no treatment	None	Median 43	86	TSH, FT4, US: anteroposterior diameter of each lobe, echogenicity	12
Karanikas [17]	Austria	*N* = 36 (18/18)	200 μg Na_2_SeO_3_ vs. placebo	All	Mean 47	100	SF-12	3
Duntas [16]	Greece	*N* = 65 (34/31)	200 μg Seme + LT4 vs. placebo + LT4	All	Mean 47.8	86	Evaluation of sleep, mood, fatigue, behavior and tiredness	6
Gartner [37]^b^	Germany	*N* = 47 (22/25)	Se-Se, Se-0, placebo-Se, placebo-0	All	Mean 41	100	US: echogenicity	6
Gartner [36]	Germany	*N* = 70 (36/24)	200 μg/d Na_2_SeO_3_ vs. placebo	All	Mean 42.3	100	US: echogenicity, SF-12	3

*int*/*con* intervention group/control group, *LT4 baseline* trial participants receiving levothyroxine (LT4) treatment at baseline, *% Fem* percentage of females in trial population, *Seme* selenomethionine, *Na*_*2*_ *SeO*_*3*_ sodium selenite, *TSH* thyroid-stimulating hormone, *FT3* free triiodothyronine, *FT4* free thyroxine, *SF-36* 36-item short-form health survey, *SF-12* 12-item short-form health survey

^a^ This trial had four intervention groups, and only the intervention groups receiving selenomethionine or placebo were included in the meta-analyses

^b^ This trial was an open-label follow-up study after that reported in ref. [36], in which participants followed different intervention regimes

#### Change in thyroid function

Two trials provided detailed information about changes in thyroid function in LT4-untreated patients [15, 39]. Eskes et al. [42] reported that 2 of 30 patients in the control and 2 of 31 patients in the intervention group, developed subclinical hypothyroidism during the months of follow-up. Pilli et al. [20] reported subclinical hypothyroidism in 2 of 20 patients in the control group vs. 2 of 40 patients in the selenium groups. The difference between selenium and control groups was not significant (RR 0.72; 95 % CI 0.19–2.75, *p* = 0.63). The quality of evidence was assessed as moderate (Tables 2 and 3). Further, five trials reported TSH levels as a continuous outcome [20, 38, 40–42], evaluated below in a meta-analysis. Meta-analyses for effects on serum thyroxine or triiodothyronine could not be performed due to heterogeneous reporting.

**Table 2:** GRADE evidence profile

Quality assessment						Quality of evidence
Outcome (number of trials)	Limitations	Inconsistency	Indirectness	Imprecision	Publication bias	
Reduction in thyroid volume assessed by ultrasound (2)	Serious limitations	No serious inconsistency	No serious indirectness	Serious imprecision (small sample size)	Undetected	++Low
Improvement in thyroid ultrasound echogenicity, LT4-untreated patients (2)	No serious limitations	Serious inconsistency (conflicting findings)	Serious indirectness (surrogate outcome)	Serious imprecision (small sample size)	Undetected	+Very low
Improvement in thyroid ultrasound echogenicity, LT4-treated patients (2)	Serious limitations	Serious inconsistency (conflicting findings)	Serious indirectness (surrogate outcome)	Serious imprecision (small sample size)	Undetected	+Very low
Improvement in HRQL, SF-36 (2)	No serious limitations	No serious inconsistency	No serious indirectness	Serious imprecision (small sample size)	Undetected	+++Moderate
Improvement in HRQL, other instruments (3)	Serious limitations (unclear blinding)	No serious inconsistency	No serious indirectness	Serious imprecision (small sample size)	Undetected	++Low
Change in thyroid function (2)^a^	No serious limitations	No serious inconsistency	No serious indirectness	Serious imprecision (small sample size)	Undetected	+++Moderate
TSH at 3 months (4)	No serious limitations	No serious inconsistency	Serious indirectness (surrogate outcome)	Serious imprecision (overall CI across 0)	Undetected	++Low
TSH at 6 months (5)	No serious limitations	No serious inconsistency	Serious indirectness (surrogate outcome)	Serious imprecision (overall CI across 0)	Undetected	++Low
TSH at 12 months (2)	No serious limitations	Serious inconsistency (heterogeneity)	Serious indirectness (surrogate outcome)	Serious imprecision (overall CI across 0)	Undetected	+Very low
Adverse effects (4)	No serious limitations	No serious inconsistency	No serious indirectness	Serious imprecision (small sample size)	Undetected	+++Moderate

^a^ Defined as the development of hypothyroidism during the course of a trial

**Table 3:** Risk of bias of included trials

	

^1^ Blinding: Unclear risk of bias for ultrasound outcomes, low risk of bias for other outcomes; Selective outcome reporting:

^2^ Selective outcome reporting: High risk of bias for ultrasound outcomes (incomplete outcome reporting, including missing account for disease duration, cannot be analyzed in a meta-analysis); Low risk of bias for other outcomes

^3^ Selective outcome reporting: High risk of bias for ultrasound outcomes, because thyroid volume as assessed by thyroid ultrasound was described in methods, but the results are not reported

^4^ Selective outcome reporting: high risk of bias for Quality of Life outcome, incomplete data reporting (cannot be analyzed in a meta-analysis); Low risk of bias for other outcomes. Other bias: High risk of bias for Quality of Life outcome (unknown outcome measure), unclear risk (unclear source of funding)

^5^ Selective outcome reporting: high risk of bias for Quality of Life outcome, incomplete data reporting (cannot be analyzed in a meta-analysis); Low risk of bias for other outcomes

^6^ Selective outcome reporting: High risk of bias for Quality of Life and ultrasound outcomes (incomplete outcome reporting, cannot be analyzed in a meta-analysis), low risk of bias for other outcomes. Other bias: High risk of bias for assessment of thyroid echogenicity, which was done qualitatively

#### Change in HRQL

Five trials evaluated HRQL [16, 17, 20, 36, 42]. Two double-blinded trials used the SF-36 form [20, 42], and both reported no significant changes following six [42] or twelve [20] months of selenium supplementation in patients not treated with LT4. Two studies used the SF-12 form and found significant improvement in well-being by 200 μg/d sodium selenite for three months, as compared to placebo [17, 36]. In both trials, selenium supplementation was administered adjuvant to LT4, and results were reported as the proportion of participants experiencing improvement. Both studies were described as ‘blinded’, without specifying if participants or investigators were blinded for intervention. One trial reported “improvement of mood and sleep and less fatigue” in 25/34 (73.5 %) participants in the intervention group receiving 200 μg/d selenomethionine for 6 months, and “amelioration of behavior and tiredness” in 15/31 (48.4 %) participants in the placebo group [16]. The authors did not specify the method used or whether the study was blinded. The above three trials, assessing HRQL in LT4-treated patients, have previously been synthesized in a meta-analysis [9]. Therefore, lacking additional trials for this patient group, we performed no meta-analysis for this outcome. The quality of evidence was assessed as low in LT4-treated patients, and moderate in LT4-untreated patients (Tables 2 and 3).

#### Change from baseline in thyroid US

Of four trials, reported in five publications, evaluating US [20, 36–38, 43], one trial enrolled LT4-treated [36], and two enrolled LT4-untreated [20, 38] patients, respectively. The fourth trial enrolled both patient groups. Among LT4-treated patients, one trial found a higher proportion of “improved echogenicity” after 3 [36] and 9 months [37] of 200 μg sodium selenite daily. Results were reported in two separate publications [36, 37], and the assessment was qualitative. In LT4-untreated patients, one trial reported no change in echogenicity or thyroid volume after twelve months of 80 or 160 μg of selenomethionine supplementation daily [20]. The other trial reported increased hypoechogenicity in controls, as compared to the intervention group, after 12 months of 80 μg sodium selenite daily [38]. Both trials evaluated echogenicity by ranging gray scale pixels from black to white [15, 37], and their results are synthesized below in a meta-analysis. The fourth trial, including both patient groups, found no change in thyroid volume or echogenicity, evaluated by gray scale pixels, after 3 months of 200 μg selenomethionine daily [43]. Quality of evidence was assessed as low for thyroid volume and very low for echogenicity (Tables 2 and 3).

#### Adverse effects

Five trials did not account for adverse effects [17, 36, 38, 41, 43]. Two trials reported no adverse effects [15, 40], including no changes in blood glucose levels [20]. Eskes et al. [42] reported two cases of hair loss, equally distributed in the placebo and selenium groups, while Krysiak et al. [39] reported two cases with complaints of nausea and headache in the selenium group, and no adverse effects in the placebo group. The difference in adverse effects between selenium and control groups was not significant (RR 2.59; 95 % CI 0.27–24.65, *p* = 0.41). The quality of evidence was assessed as moderate (Tables 2 and 3).

### Meta-analysis

#### Change in TSH level

**Fig. 2:**
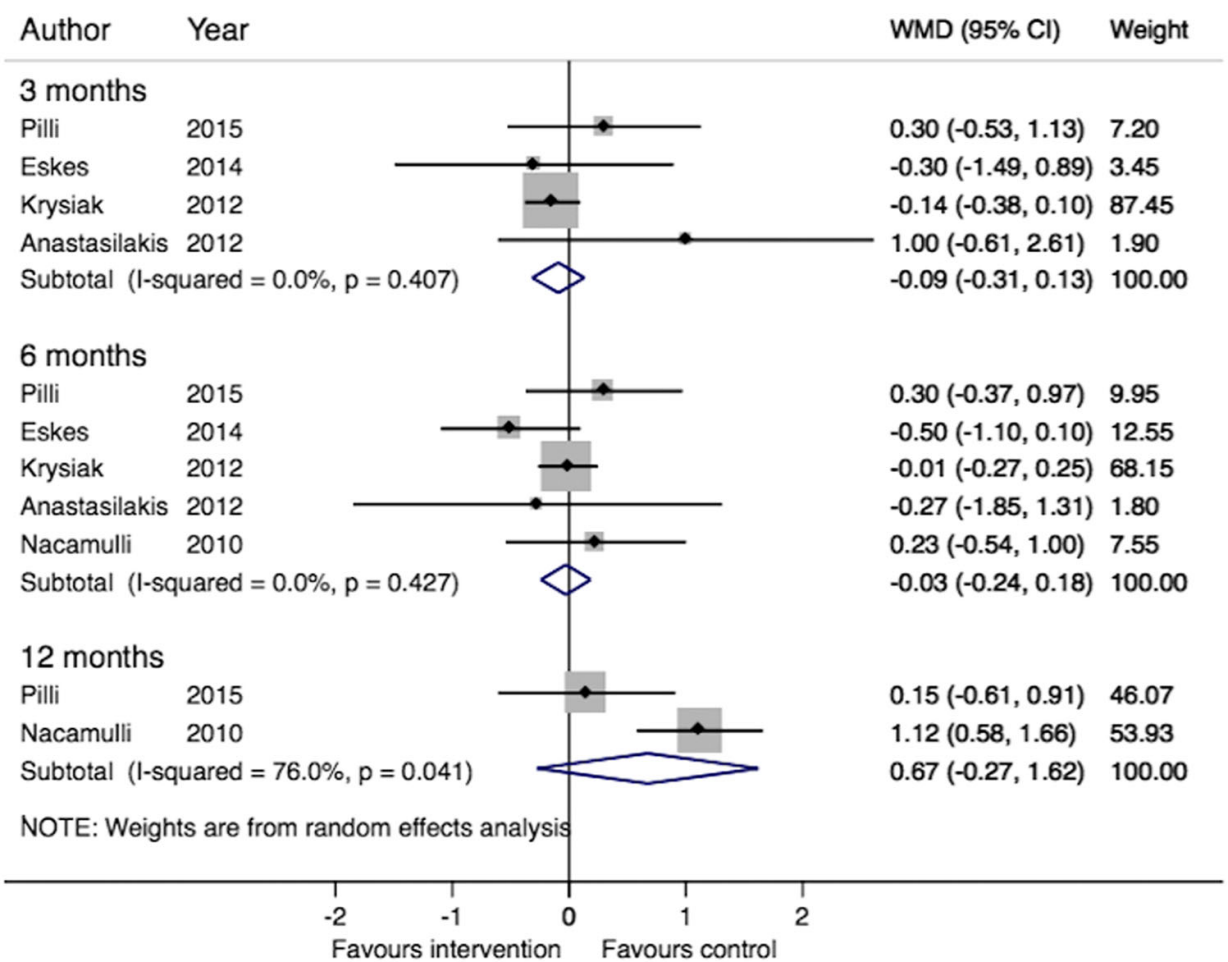
Meta-analysis of the effect of selenium supplementation on serum thyrotropin (thyroid simulating hormone) levels in populations of chronic AIT patients not receiving LT4. Weighted mean difference (WMD) in serum TSH levels after 3, 6 and 12 months’ selenium supplementation vs. control in populations of AIT patients not receiving LT4. *Boxes* represent mean values of the outcomes in a study, *horizontal lines* the 95 % confidence intervals, and the *box area* is proportional to the weight of the individual study (as seen in “Weight”). *Diamonds* represent the overall summary estimate, with confidence interval given by its width. *I* ^2^ shows the heterogeneity among studies, and with *p*-value

Compared to control groups, there was no significant change in TSH levels following selenium supplementation in LT4-untreated populations after three months (four trials, WMD: −0.09, 95 % CI −0.31–0.13, *p* = 0.42, *I* ^2^ = 0.0 %), 6 months (five trials, WMD: −0.03 95 % CI −0.24–0.18, *p* = 0.80, *I* ^2^ = 0.0 %), or 12 months (two trials, WMD 0.67, 95 % CI −0.27–1.62, *p* = 0.16, *I* ^2^ = 76.0 %). Results are illustrated in Fig. 2. The quality of evidence was assessed as low at 3 and 6 months, and very low at 12 months (Tables 2 and 3).

#### Change in HRQL (SF-36)

Compared to control groups, there were no significant changes in any of the eight SF-36 domains following 6 months of selenium supplementation in two trials enrolling LT4-untreated individuals (data not shown) [20, 42]. The quality of evidence was assessed as moderate (Tables 2 and 3)

#### Change in thyroid echogenicity

Compared to control groups, there were no significant improvements in thyroid echogenicity following six (two trials, WMD 2.88, 95 % CI −4.50–10.25, *p* = 0.45, *I* ^2^ = 57.7 %) or twelve (two, trials, WMD 1.92, 95 % CI: −4.22–8.06, *p* = 0.54, *I* ^2^ = 46.5 %) months of selenium supplementation in two trials enrolling LT4-untreated individuals [20, 38]. The quality of evidence was assessed as very low (Tables 2 and 3)

### Quality of evidence assessment

Quality of evidence was assessed per outcome (Tables 2 and 3), using the GRADE guidelines [19]. For outcomes not evaluated in meta-analyses, inconsistency was assessed by comparing trial findings. Thyroid echogenicity and TSH levels were downgraded for indirectness, since we deemed these outcomes surrogate markers of lymphocytic infiltration and disease progression, respectively [13]. Egger’s test (funnel plot) could not be performed, since none of our meta-analyses contained ten studies or more [15]. However, assessing if smaller trials were more prone to reporting positive results than larger ones, showed no sign of publication bias.

## Discussion

A key aspect in our evaluation of the nine included trials was the division into populations of LT4-treated and -untreated patients. This was done, according to GRADE recommendations [13], because the outcomes deemed important a priori were different in the respective populations. Three of the included trials enrolled patients receiving LT4 [16, 17, 36], four trials dealt exclusively with untreated patients [20, 38, 39, 42], and two trials included both untreated and treated patients [41, 43].

In the three trials where selenium was administered adjuvant to LT4, thyroid function could not readily be assessed as an outcome due to the impact of LT4 per se. Mortality, morbidity and disease remission are rated as crucial according to the GRADE recommendations, and of obvious concern, since hypothyroidism is associated with increased somatic [44] and psychiatric morbidity [45], as well as excess mortality [46]. However, these endpoints are relatively infrequent, occur over longer periods of time [13], and were not assessed in any of the identified trials. LT4 dose and/or HRQL constitute more readily assessable relevant outcomes. No previous trials reported LT4 dose as an outcome, but data on HRQL [33, 35, 36] have previously been synthesized into meta-analyses [9, 10]. One reported that patients assigned to selenium supplementation experienced improved well-being or mood, as compared with controls [9], and the other study showed no change [10]. However, neither of the previous reviews assessed the quality of evidence, which we consider to be low due to unclear blinding in relation to this subjective outcome. Furthermore, none used a validated HRQL instrument for hypothyroid patients, such as the ThyPRO [47, 48]. By assessment with this instrument we have demonstrated widely impaired HRQL, that is not normalized following six months of LT4 treatment [49]. Finally, independent effects on thyroid morphology, on top of the well-known effects of LT4 on thyroid size [50], are difficult to assess in this group because the trials [36, 43] did not account for disease duration.

In the LT4-untreated population, we prioritized disease progression as the most important outcome. It can be evaluated categorically by the proportion of patients developing subclinical or overt hypothyroidism during the time of intervention or follow-up. Two studies reported direct data for this aspect [20, 42], showing no effect. In addition, our meta-analysis for effects on TSH levels, reached the same conclusion. Two trials assessed HRQL effects [20, 42], and we found no significant effects in a meta-analysis on any SF-36 domains. While HRQL is always an outcome of some importance, it might be of limited relevance in untreated euthyroid patients, who are more likely to be unaffected than are hypothyroid treated patients. Two trials also investigated thyroid volume and echogenicity quantitatively [20, 38]. The studies yielded contradictory results for echogenicity, but when synthesized in a meta-analysis, the studies showed no effect.

Selenium supplementation did not significantly impact the incidence of adverse effects, which were reported only in two double-blinded trials [39, 42]. Evaluation of long-term morbidity following trial participation, would address recently voiced safety concerns [14], e.g. regarding the implications of selenium supplementation for glucose metabolism. This issue was evaluated in only one trial and found blood glucose levels unaltered [20].

Interestingly, direct clinical outcomes have been assessed in another thyroid patient group. In a randomized placebo-controlled trial, 169 euthyroid TPO-Ab positive pregnant women were allocated to 200 μg/d selenomethionine or matching placebo from 12 weeks gestation to 12 months after delivery [28]. Post partum thyroid dysfunction and permanent hypothyroidism were significantly less prevalent in patients receiving selenium, as compared to placebo. Based on this, the authors concluded that selenium supplementation reduced thyroid inflammatory activity and the incidence of hypothyroidism in the post partum phase [28].

Seven of the included nine trials reported selenium status in their patients. Six European trial populations [15, 33, 35, 36, 38, 39] were within a narrow range (70–85 μg/L) of baseline serum/plasma selenium concentrations, while it was lower (37 μg/L) in the Brazilian trial [40]. The current selenium reference range is 100–120 μg/L [51], implying marginal selenium deficiency in the European and more marked deficiency in the Brazilian participants. The nine identified trials intervened with 80–200 μg/day, using different formulations with varying bioavailablity; the absorption of selenite is approximately 2/3 of the absorption of selenomethionine [52]. In a recent narrative review it was suggested that selenomethionine might be more effective than selenite in lowering thyroid autoantibody levels [53]. However, no such trends were observed for any of the clinical outcomes assessed in our study. The duration of intervention exceeded six months only in two trials [20, 38]. Just in one trial [20] did selenium levels at the end of intervention exceed the reference range, and in the Brazilian trial [43], the plasma selenium concentration in the intervention group increased only to 63.4 μg/L. Thus, trial findings should mainly be interpreted in relation to correcting marginal deficits, rather than administering supranutrional doses.

There are limitations to our study, and while the GRADE approach rates the quality of evidence systematically, it does not eliminate judgment [54], which is a limitation per se*.* A statistical source of error is that one study presented data in median with IQR [20] and another in median with range [42], both of which may be unreliable for calculating a mean and a SD for the meta-analysis [20]. However, excluding these studies [20, 42] had no influence on the results. Finally, the meta-analysis were performed on the basis on very few studies, limiting the quality of the evidence.

Future trials of selenium supplementation in AIT should pay close attention to the selenium status of the study populations, and identify outcomes of clinical importance relating to the eligibility criteria set for trial participants. In euthyroid individuals, whose tolerance to TPO or thyroglobulin is broken, but where clinical disease is absent, we recommend disease progression as an important outcome. However, we found no effects on TSH level, which may be considered as a surrogate marker, or on HRQL or thyroid echogenicity. In hypothyroid patients, a rigorous trial investigating LT4-dose titration as an outcome could unmask implications for disease remission. Finally, although the quality of evidence is low, previous trial results give some promise of beneficial effects on HRQL. This outcome can now be evaluated using validated disease-specific instruments [47, 48, 55], one of which (ThyPRO) is the primary outcome in an ongoing trial [56].

There have been conflicting reports as to whether selenium supplementation is of benefit in patients with AIT [53]. Our conclusion is that current evidence does not justify the emerging use of selenium supplementation in the treatment of AIT [57]. While the correction of a selenium deficit may offer other health benefits [8], routine selenium supplementationin AIT patients should, at present, be discouraged [58].

## References

[1] Carle A, Laurberg P, Pedersen IB, Knudsen N, Perrild H, Ovesen L, Rasmussen LB, Jorgensen T (2006). Epidemiology of subtypes of hypothyroidism in Denmark. Eur J Endocrinol..

[2] Vanderpump MP (2011). The epidemiology of thyroid disease. Br Med Bull..

[3] Brix TH, Kyvik KO, Hegedus L (2000). A population-based study of chronic autoimmune hypothyroidism in Danish twins. J Clin Endocrinol Metab..

[4] Brix TH, Hegedus L (2012). Twin studies as a model for exploring the aetiology of autoimmune thyroid disease. Clin Endocrinol..

[5] Wu Q, Rayman MP, Lv H, Schomburg L, Cui B, Gao C, Chen P, Zhuang G, Zhang Z, Peng X, Li H, Zhao Y, He X, Zeng G, Qin F, Hou P, Shi B (2015). Low population selenium status is associated with increased prevalence of thyroid disease. J Clin Endocrinol Metab..

[6] Wiersinga WM (2014). Paradigm shifts in thyroid hormone replacement therapies for hypothyroidism. Nat Rev Endocrinol.

[7] Schomburg L (2012). Selenium, selenoproteins and the thyroid gland: interactions in health and disease. Nat Rev Endocrinol.

[8] Rayman MP (2012). Selenium and human health. Lancet.

[9] Toulis KA, Anastasilakis AD, Tzellos TG, Goulis DG, Kouvelas D (2010). Selenium supplementation in the treatment of Hashimoto’s thyroiditis: a systematic review and a meta-analysis. Thyroid.

[10] Fan Y, Xu S, Zhang H, Cao W, Wang K, Chen G, Di H, Cao M, Liu C (2014). Selenium supplementation for autoimmune thyroiditis: a systematic review and meta-analysis. Int J Endocrinol..

[11] van Zuuren Esther J, Albusta Amira Y, Fedorowicz Z, Carter B, Pijl H (2013). Selenium supplementation for Hashimoto’s thyroiditis. Cochrane Database Syst Rev..

[12] Jensen EA, Petersen PH, Blaabjerg O, Hansen PS, Brix TH, Hegedus L (2006). Establishment of reference distributions and decision values for thyroid antibodies against thyroid peroxidase (TPOAb), thyroglobulin (TgAb) and the thyrotropin receptor (TRAb). Clin Chem Lab Med..

[13] Guyatt GH, Oxman AD, Kunz R, Atkins D, Brozek J, Vist G, Alderson P, Glasziou P, Falck-Ytter Y, Schunemann HJ (2011). GRADE guidelines: 2. Framing the question and deciding on important outcomes. J Clin Epidemiol.

[14] Jablonska E, Vinceti M (2015). Selenium and human health: witnessing a Copernican revolution?. J Environ Sci Health C Environ Carcinog Ecotoxicol Rev.

[15] J.P.T. Higgins, GSe. Cochrane handbook for systematic reviews of interventions Version 5.1.0. The Cochrane Collaboration (2011), http://handbook.cochrane.org.

[16] Duntas LH, Mantzou E, Koutras DA (2003). Effects of a six month treatment with selenomethionine in patients with autoimmune thyroiditis. Eur J Endocrinol.

[17] Karanikas G, Schuetz M, Kontur S, Duan H, Kommata S, Schoen R, Antoni A, Kletter K, Dudczak R, Willheim M (2008). No immunological benefit of selenium in consecutive patients with autoimmune thyroiditis. Thyroid.

[18] Ware JE, Sherbourne CD (1992). The MOS 36-item short-form health survey (SF-36). I. Conceptual framework and item selection. Med Care.

[19] Balshem H, Helfand M, Schunemann HJ, Oxman AD, Kunz R, Brozek J, Vist GE, Falck-Ytter Y, Meerpohl J, Norris S, Guyatt GH (2011). GRADE guidelines: 3. Rating the quality of evidence. J Clin Epidemiol.

[20] Pilli T, Cantara S, Schomburg L, Cenci V, Cardinale S, Heid EC, Kuhn EC, Cevenini G, Sestini F, Fioravanti C, D’Hauw G, Pacini F (2015). IFNgamma-inducible chemokines decrease upon selenomethionine supplementation in women with euthyroid autoimmune thyroiditis: comparison between two doses of selenomethionine (80 or 160 mug) versus placebo. Eur Thyroid J..

[21] Balazs C (2008). The effect of selenium therapy on autoimmune thyroiditis. Orv Hetil.

[22] Cenci V, Pilli T, Cardinale S (2013). Selenomethionine supplementation in euthyroid patients with autoimmune thyroiditis. Eur Thyroid J..

[23] S. Ciric Effects of selenium supplementation on TPOAb in active autoimmune thyroiditis. Eur. Thyroid J. **1,** 163 (2011)

[24] Duntas L, Boutsiadis A, Mantzou E (2013). Selenium supplementation in pregnant women with chronic autoimmune thyroiditis: effects on selenium, serum concentration and autoimmune parameters. Eur Thyroid J..

[25] Guglielmi R, Misischi I, Graziano F (2013). Selenium supplementation in euthyroid patients with autoimmune thyroiditis. A pilot controlled study. Eur Thyroid J..

[26] Kvicala J, Hrda P, Zamrazil V (2010). Affects selenium indeed concentrations of serum autoantibodies TPOAb and TGAb?. Trace Elem Electrolyt.

[27] Zhu L, Bai X, Teng WP, Shan ZY, Wang WW, Fan CL (2012). Effects of selenium supplementation on antibodies of autoimmune thyroiditis. Zhonghua Yi Xue Za Zhi..

[28] Negro R, Greco G, Mangieri T, Pezzarossa A, Dazzi D, Hassan H (2007). The influence of selenium supplementation on postpartum thyroid status in pregnant women with thyroid peroxidase autoantibodies. J Clin Endocrinol Metab..

[29] Mao J, Pop VJ, Bath SC, Vader HL, Redman CW, Rayman MP (2016). Effect of low-dose selenium on thyroid autoimmunity and thyroid function in UK pregnant women with mild-to-moderate iodine deficiency. Eur J Nutr..

[30] Nordio M, Pajalich R (2013). Combined treatment with Myo-inositol and selenium ensures euthyroidism in subclinical hypothyroidism patients with autoimmune thyroiditis. J Thyroid Res..

[31] Zagrodzki P, Ratajczak R (2008). Selenium supplementation in autoimmune thyroiditis female patient--effects on thyroid and ovarian functions (case study). Biol Trace Elem Res.

[32] Turker O, Kumanlioglu K, Karapolat I, Dogan I (2006). Selenium treatment in autoimmune thyroiditis: 9-month follow-up with variable doses. J Endocrinol..

[33] Mazokopakis EE, Papadakis JA, Papadomanolaki MG, Batistakis AG, Giannakopoulos TG, Protopapadakis EE, Ganotakis ES (2007). Effects of 12 months treatment with L-selenomethionine on serum anti-TPO Levels in Patients with Hashimoto’s thyroiditis. Thyroid..

[34] Kvicala J, Hrda P, Zamrazil V, Nemecek J, Hill M, Jiranek V (2009). Effect of selenium supplementation on thyroid antibodies. J Radioanalyt Nucl Chem..

[35] Bhuyan AK, Sarma D, Saikia UK (2012). Selenium and the thyroid: A close-knit connection. Indian J Endocrinol Metab..

[36] Gartner R, Gasnier BC, Dietrich JW, Krebs B, Angstwurm MW (2002). Selenium supplementation in patients with autoimmune thyroiditis decreases thyroid peroxidase antibodies concentrations. J Clin Endocrinol Metab..

[37] Gartner R, Gasnier BC (2003). Selenium in the treatment of autoimmune thyroiditis. Biofactors..

[38] Nacamulli D, Mian C, Petricca D, Lazzarotto F, Barollo S, Pozza D, Masiero S, Faggian D, Plebani M, Girelli ME, Mantero F, Betterle C (2010). Influence of physiological dietary selenium supplementation on the natural course of autoimmune thyroiditis. Clin Endocrinol..

[39] Krysiak R, Okopien B (2011). The effect of levothyroxine and selenomethionine on lymphocyte and monocyte cytokine release in women with Hashimoto’s thyroiditis. J Clin Endocrinol Metab..

[40] Krysiak R, Okopien B (2012). Haemostatic effects of levothyroxine and selenomethionine in euthyroid patients with Hashimoto’s thyroiditis. Thromb Haemost.

[41] Anastasilakis AD, Toulis KA, Nisianakis P, Goulis DG, Kampas L, Valeri RM, Oikonomou D, Tzellos TG, Delaroudis S (2012). Selenomethionine treatment in patients with autoimmune thyroiditis: a prospective, quasi-randomised trial. Int J Clin Pract..

[42] Eskes SA, Endert E, Fliers E, Birnie E, Hollenbach B, Schomburg L, Kohrle J, Wiersinga WM (2014). Selenite supplementation in euthyroid subjects with thyroid peroxidase antibodies. Clin Endocrinol.

[43] de Farias CR, Cardoso BR, de Oliveira GM, de Mello Guazzelli IC, Catarino RM, Chammas MC, Cozzolino SM, Knobel M (2015). A randomized-controlled, double-blind study of the impact of selenium supplementation on thyroid autoimmunity and inflammation with focus on the GPx1 genotypes. J Endocrinol Invest.

[44] Thvilum M, Brandt F, Almind D, Christensen K, Brix TH, Hegedus L (2013). Type and extent of somatic morbidity before and after the diagnosis of hypothyroidism. A nationwide register study. PLoS One.

[45] Thvilum M, Brandt F, Almind D, Christensen K, Brix TH, Hegedus L (2014). Increased psychiatric morbidity before and after the diagnosis of hypothyroidism: a nationwide register study. Thyroid.

[46] Thvilum M, Brandt F, Almind D, Christensen K, Hegedus L, Brix TH (2013). Excess mortality in patients diagnosed with hypothyroidism: a nationwide cohort study of singletons and twins. J Clin Endocrinol Metab..

[47] Watt T, Cramon P, Hegedus L, Bjorner JB, Bonnema SJ, Rasmussen AK, Feldt-Rasmussen U, Groenvold M (2014). The thyroid-related quality of life measure ThyPRO has good responsiveness and ability to detect relevant treatment effects. J Clin Endocrinol Metab.

[48] Watt T, Bjorner JB, Groenvold M, Cramon P, Winther KH, Hegedus L, Bonnema SJ, Rasmussen AK, Ware JE, Feldt-Rasmussen U (2015). Development of a short version of the thyroid-related patient-reported outcome ThyPRO. Thyroid.

[49] Winther KH, Cramon P, Watt T, Bjorner JB, Ekholm O, Feldt-Rasmussen U, Groenvold M, Rasmussen ÅK, Hegedüs L, Bonnema SJ (2016). Disease-specific as well as generic quality of life is widely impacted in autoimmune hypothyroidism and improves during the first six months of levothyroxine therapy. PLoS One.

[50] Hegedus L, Hansen JM, Feldt-Rasmussen U, Hansen BM, Hoier-Madsen M (1991). Influence of thyroxine treatment on thyroid size and anti-thyroid peroxidase antibodies in Hashimoto’s thyroiditis. Clin Endocrinol.

[51] Kipp AP, Strohm D, Brigelius-Flohe R, Schomburg L, Bechthold A, Leschik-Bonnet E, Heseker H (2015). Revised reference values for selenium intake. J Trace Elem Med Biol.

[52] Burk RF, Norsworthy BK, Hill KE, Motley AK, Byrne DW (2006). Effects of chemical form of selenium on plasma biomarkers in a high-dose human supplementation trial. Cancer Epidemiol Biomarkers Prev.

[53] Duntas LH, Benvenga S (2015). Selenium: an element for life. Endocrine.

[54] Guyatt G, Oxman AD, Akl EA, Kunz R, Vist G, Brozek J, Norris S, Falck-Ytter Y, Glasziou P, DeBeer H, Jaeschke R, Rind D, Meerpohl J, Dahm P, Schunemann HJ (2011). GRADE guidelines: 1. Introduction-GRADE evidence profiles and summary of findings tables. J Clin Epidemiol.

[55] C.K. Wong, B.H. Lang, C.L. Lam: A systematic review of quality of thyroid-specific health-related quality-of-life instruments recommends ThyPRO for patients with benign thyroid diseases. J. Clin. Epidemiol. (2016). doi:10.1016/j.jclinepi.2016.03.006 10.1016/j.jclinepi.2016.03.006 27020087

[56] Winther KH, Watt T, Bjorner JB, Cramon P, Feldt-Rasmussen U, Gluud C, Gram J, Groenvold M, Hegedus L, Knudsen N, Rasmussen AK, Bonnema SJ (2014). The chronic autoimmune thyroiditis quality of life selenium trial (CATALYST): study protocol for a randomized controlled trial. Trials.

[57] R. Negro, R. Attanasio, F. Grimaldi, C. Marcocci, R. Guglielmi, E. Papini, A 2016 Italian Survey about the clinical use of selenium in thyroid disease. Eur. Thyroid J., in press (2016) 10.1159/000447667 PMC5091264 27843806

[58] L. Hegedüs, S.J. Bonnema, K.H. Winther, Selenium in the treatment of thyroid diseases. An element in search of the relevant indications? Eur. Thyroid J., in press (2016). doi: 10.1159/000448002 10.1159/000448002 PMC5091242 27843804

